# A study of assessments of camptocormia

**DOI:** 10.1186/1748-7161-8-S1-O31

**Published:** 2013-06-03

**Authors:** M De Sèze, E Guillaud, L Slugatz, JR Cazalets

**Affiliations:** 1 CHU de Bordeaux, Université Bordeaux Ségalen, Bordeaux, France

## Introduction

Camptocormia (or bent spine syndrome) is defined as abnormal posture, with marked flexion of the thoracolumbar spine, increasing during walking, and disappearing in the supine position. This definition is not so clear, and indeed, there are no consensus criteria for diagnosing camptocormia. Consequently, the prevalence of camptocormia is difficult to determine. More often, the diagnosis is made by subjectively assessing the patient’s posture, but this approach is no more satisfying, because the camptocormia can be overestimated or underestimated.

## Aim

The aim of our study was to investigate different methods for assessing camptocormia.

## Material and methods

Camptocormia was subjectively defined as forward flexion of the thoracolumbar spine that disappeared in the recumbent position. Patients were excluded if they were not able to walk more than ten meters without human or technical help. All the includable patients were convoked one afternoon for clinical, radiological, and kinematical assessments for the following 30 days. Parameters: Two visual radiological and kinematic markers were initially positioned on the patients. They were left on the skin during all the assessment, in order to be able to relay clinical, radiological and kinematical conditions. One marker was positioned in regard to the spinous process of C7, and the other marker was positioned in the middle of the segment relaying the two Michaëlis fovea, in order to project horizontally to S1. Sagittal inclinations were valued by the C7 arrow, defined as the distance from the C7 marker to a plumb line of the S1 marker.

## Results

Forty three patients (28 females and 15 males; 69 ± 10 years) were included in the study. The mean increase of the C7 arrow during walking was 64.2 ± 45.6 mm for the patients presenting an initial C7 arrow superior or equal to 200 mm, and was 31.6 ± 30.1 mm for the patients presenting an initial C7 arrow inferior to 200 mm, (p= 0.0040). For each static condition, we could define a similar threshold separating the patients in two groups of and increasing C7 arrow during walking.

## Conclusion

These results open perspectives in order to propose objective definition of camptocormia.
